# Cytomegalovirus as an Uninvited Guest in the Response to Vaccines in People Living with HIV

**DOI:** 10.3390/v13071266

**Published:** 2021-06-29

**Authors:** Léna Royston, Stéphane Isnard, John Lin, Jean-Pierre Routy

**Affiliations:** 1 Infectious Diseases and Immunity in Global Health Program, Research Institute, McGill University Health Centre, Montréal, QC H4A 3J1, Canada; lena.royston@mail.mcgill.ca (L.R.); stephane.isnard@mail.mcgill.ca (S.I.); john.lin@mail.mcgill.ca (J.L.); 2 Chronic Viral Illness Service, McGill University Health Centre, Montréal, QC H4A 3J1, Canada; 3 Canadian Institutes of Health Research, Canadian HIV Trials Network, Vancouver, BC V6Z 1Y6, Canada; 4 Division of Hematology, McGill University Health Centre, Montréal, QC H4A 3J1, Canada

**Keywords:** HIV, cytomegalovirus, CMV, vaccine, immunosenescence, immune activation, gut inflammation

## Abstract

In stark contrast to the rapid development of vaccines against severe acute respiratory syndrome coronavirus 2 (SARS-CoV-2), an effective human immunodeficiency virus (HIV) vaccine is still lacking. Furthermore, despite virologic suppression and CD4 T-cell count normalization with antiretroviral therapy (ART), people living with HIV (PLWH) still exhibit increased morbidity and mortality compared to the general population. Such differences in health outcomes are related to higher risk behaviors, but also to HIV-related immune activation and viral coinfections. Among these coinfections, cytomegalovirus (CMV) latent infection is a well-known inducer of long-term immune dysregulation. Cytomegalovirus contributes to the persistent immune activation in PLWH receiving ART by directly skewing immune response toward itself, and by increasing immune activation through modification of the gut microbiota and microbial translocation. In addition, through induction of immunosenescence, CMV has been associated with a decreased response to infections and vaccines. This review provides a comprehensive overview of the influence of CMV on the immune system, the mechanisms underlying a reduced response to vaccines, and discuss new therapeutic advances targeting CMV that could be used to improve vaccine response in PLWH.

## 1. Introduction

The development of antiretroviral therapy (ART) against human immunodeficiency virus (HIV) has dramatically transformed the lives of people living with HIV (PLWH) and turned a life-threatening infection into a manageable, yet chronic, disease [1]. However, despite maintaining undetectable plasma HIV viral load, ART is still unable to eradicate HIV as the virus hides in proviral reservoirs. In 2020, 40 years into the HIV epidemic, 38 million people were living with HIV, including 1.7 million newly-infected individuals [2]. An efficient preventive vaccine could hamper transmission of the virus and curb the HIV epidemic. However, compared to the rapid development of vaccines against severe acute respiratory syndrome coronavirus 2 (SARS-CoV-2), an HIV vaccine is still lacking [1]. An optimal immune response to HIV is limited by different factors that include: (1) the unequaled genetic diversity and mutation rate of HIV; (2) the ability to infect and repress the very cells that orchestrate immune response, the CD4 helper T-cells; (3) the ability of the virus to integrate in the host genome and evade the immune response; (4) the persistence of immune activation despite long-term ART; and (5) the influence of other chronic viral coinfections, like cytomegalovirus (CMV) that exacerbate HIV-induced immune dysregulation. In this review, we will focus on the influence of CMV on vaccine response and development of anti-HIV vaccines, since CMV almost universally co-infects PLWH and has been associated with enhanced immune activation. We aim to provide a comprehensive review of mechanisms by which CMV shapes the whole immune system, notably through the enhancement of gut microbial translocation, which contributes to reduced vaccine responses in PLWH.

## 2. Human Immunodeficiency Virus (HIV) Vaccine Development: Impact of Viral-Induced Immune Activation

The greatest challenge encountered in the development of HIV vaccines has been the high mutation and recombination rates of the HIV genome during viral replication [3]. The viral envelope (Env) glycoprotein is the main driver of HIV genetic diversity, while constituting at the same time the major target of neutralizing antibodies. Despite this tremendous diversity, five HIV vaccines have reached large-scale phase III efficacy trials. The most promising study remains RV144, with a modest but significant efficacy of 31% against HIV-1 infection. This Thai study investigated a vaccine regimen containing the ALVAC-HIV canarypox vector-based vaccine, boosted with a two-component gp120 protein subunit vaccine AIDSVAX^®^B/E [4]. However, the hope faded with the results of the confirmation study HIV Vaccine Trials Network (HVTN) 702 conducted in South Africa using the same prime-boost vaccine regimen on HIV clade C, which was prematurely halted as it met the prespecified stopping criteria for non-efficacy [5]. The discovery in the sera of PLWH of broadly neutralizing antibodies now provides new clues to design effective vaccine candidates [6,7].

Although the advent of ART significantly improved the magnitude and duration of immune response to vaccination in PLWH, immune activation that persists despite effective ART alters the response to vaccines. A study by George et al. evaluated the immune response to seasonal influenza vaccination in PLWH compared to age-matched controls [8]. Interestingly, specific antibody-secreting cell responses was similar in old HIV-uninfected participants and in young PLWH participants, suggesting an acceleration or enhanced immune senescence with HIV infection. Moreover, non-responsiveness was correlated with the level of CD4 T-cells activation and Tumor Necrosis Factor (TNF)-α plasma levels.

Aside from HIV-induced immune activation, interindividual variations in vaccine response have been linked to pre-immune T-cell repertoire diversity, driven by previous infections and vaccinations, or gut microbiome composition and microbial translocation. As an example, a study reported an association between the composition of gut microbiota and the HIV-1 immune responses to the NYVAC vaccine candidate in the HVTN 096 trial participants [9]. Although many other factors might be involved, the discrepancy of results between HVTN 702 and the previous RV144 trial, which were conducted in various environments, could also be explained by differences in preexisting exposure to diseases and microbiome disparities.

Every actor shaping the immune system should thus be studied in order to increase the likelihood of success for future HIV vaccines. Those factors notably include CMV, a well-known modulator of the human immune response.

## 3. The Direct Effect of Cytomegalovirus (CMV) on the Immune System

**Figure 1:**
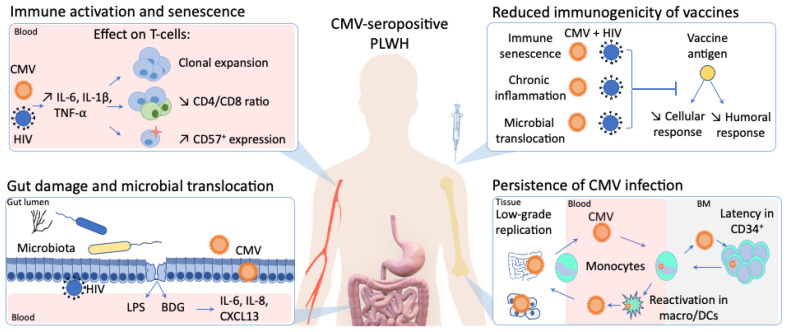
The influence of cytomegalovirus (CMV) persistent infection on immune activation, immunogenicity of vaccines, gut inflammation in people living with human immunodeficiency virus (HIV).

Diversity of the human immune system is considerable and contributes to interindividual differential responses to pathogens and vaccines. Viruses have co-evolved with humans for millennia and are known to modulate the immune response and to contribute to those variations. Member of the Betaherpesvirinae subfamily of the Herpesvirales order and assigned to the Duplodnaviria realm by the International Committee on Taxonomy of Viruses (ICTV) in 2020 [10], CMV evolved a complex arsenal to manipulate the immune system of its host. Up to 70–90% of humans worldwide are infected during their lifespan and risk factors for seropositivity include age, sex, race, geographical regions and household income [11,12]. Cytomegalovirus primary infection is usually asympto- or pauci-symptomatic in immunocompetent hosts. After primary mucosal CMV infection, infected monocytes infiltrate the bone marrow where it establishes latency in a small subset of pluripotent CD34+ hematopoietic stem cells (HSC) (Figure 1) [13]. During CMV reactivation events, blood-borne monocytes derived from infected CD34+ HSCs will in turn allow viral spread while differentiating into dendritic cells and macrophages, inducing CMV lytic gene expression and reinfection of epithelial cells, as in the gastrointestinal tract [14,15]. Frequent episodes of subclinical reactivations, usually triggered by biological stress, force the immune system to adapt in order to restrain this replication and to prevent life-threatening infections. Reports suggest that higher levels of anti-CMV specific IgG reflect frequent episodes of such reactivation events [16,17]. Recent data also suggest that genetic variability in killer immunoglobulin-like receptor (KIR) repertoire of NK cells, as well as in IgG genes, may affect the control of CMV infection [18,19].

The influence of CMV on the development of the immune system is epitomized in monozygotic twin studies, in which serodiscordance for CMV was found to influence 58% of all measured immune parameters [20]. Cytomegalovirus seropositivity has been extensively associated with CD8 T-cell expansion, and a decreased CD4/CD8 T-cell ratio [17,21]. After years of latent infection, up to 30% and 50% of effector memory CD4 and CD8 T-cells, respectively, target CMV antigens specifically [22]. These CMV-specific T-cells have been shown to exhibit senescence phenotypic characteristics, such as shortened telomeres, decreased expression of fitness markers CD27 and CD28 and increased expression of senescence marker CD57 [23].

CMV chronic infection has also been shown to generate low-grade inflammation which contributes to “inflammaging” which describes an increase in pro-inflammatory cytokine levels that occurs with older age [24].

In PLWH, CMV seropositivity is almost universal and potentiates HIV-induced immune activation, further contributing to non-AIDS comorbidities. Asymptomatic CMV shedding is frequently detected in the blood and the genital tract of PLWH, and has been associated with T-cell immune activation and a decreased CD4/CD8 T-cell ratio (Figure 1) [17,25].

## 4. CMV as a Perturbator of Gut Barrier and Microbiota in People Living with HIV (PLWH)

### 4.1. Gut as a Viral Sanctuary

Many viruses chronically infect the gut mucosa and constitute the gut virome [26]. During both acute and chronic phases of HIV infection, the gut contains a large number of infected cells, due to local T-cell activation and high C-C chemokine receptor (CCR)-5 expression [27]. Depletion of mucosal CD4 T-cells upon HIV infection impairs the gut barrier integrity and leads to microbial translocation and microbiota changes [28,29]. However, despite effective ART and T-cell restoration, gut permeability and dysbiosis remain in PLWH and have been associated with systemic immune activation and non-AIDS comorbidities [30,31]. In addition, as the largest lymphoid organ, the gut constitutes a considerable reservoir for HIV, with low distribution of ART to this compartment [32]. Regarding CMV, symptomatic colitis presents only in the case of severe immunodeficiency. However, asymptomatic CMV detection in the gut mucosa has also been reported both in HIV-uninfected people and ART-treated PLWH [14,15,33].

### 4.2. Gut Damage and Microbial Translocation

Constantly in contact with nutrients, commensal microbes and invading pathogens, the gut barrier plays a complex role in allowing nutrient absorption while battling against microbe translocation.

Damage to the gastrointestinal epithelial gut barrier and subsequent translocation of microbes and their byproducts in the circulation constitute hallmarks of HIV infection and participate in systemic inflammation during chronic HIV infection [28,34,35]. The exact mechanisms responsible for gut damage and epithelial permeability are not fully understood, and these alterations do not improve upon ART initiation. Early infection and depletion of gut-associated lymphoid tissue (GALT) resident CD4 T-cells is associated with early HIV-induced enteropathy [36,37]. In addition, HIV-1 exposure on intestinal mucosa was shown to directly induce inflammatory cytokine production like TNF-α [38] and Interleukin 18 (IL-18) [39] from epithelial cells, further disrupting tight-junction proteins between epithelial cells. Although GALT is a well-described HIV reservoir, HIV persistence does not fully explain the persistence of gut damage in ART-treated PLWH.

A study analyzing gut biopsies of 19 ART-treated PLWH showed that CMV detection was associated with the disrupted epithelial barrier and decreased zonula occludens-1 (ZO-1) expression, a marker of tight junctions [14]. We have recently reported in a cross-sectional study involving long term ART-treated PLWH that CMV seropositivity was associated with persistent elevated CD8 T-cell counts and lower CD4/CD8 ratio [17]. In this study, CMV seropositivity was associated with higher plasma levels of gut damage markers (intestinal fatty-acid binding protein [I-FABP]) and microbial translocation (lipopolysaccharide [LPS], β-d-Glucan [BDG]) in both PLWH and HIV-uninfected participants [17]. In addition, this gut leakage resulted in an increase of pro-inflammatory cytokines (CXCL13, IL-6, IL-8) only in PLWH. A correlation between gut damage/microbial translocation markers and anti-CMV IgG levels was also found, conversely to the absence of correlation with levels of anti-EBV IgG or total IgG, IgM, and IgA.

### 4.3. Gut Microbiota

Mounting evidence have associated gut microbiota, including the bacterial communities but also the virome, with host metabolism and inflammation [26,40]. In addition, the gut microbiota has also emerged as a central player in the development and modulation of the immune system, notably through microbial by-products that include small-chain fatty acids such as acetate, propionate, and butyrate [41,42]. Those short chain fatty acids, which are produced by commensal bacteria, act as signaling molecules on epithelial and immune cells and regulate cytokine production of T-cells and regulatory T cells (Tregs) promotion [43,44]. In addition to these direct effects, other mechanisms of immune regulation by microbiota have been proposed such as modulation of the activation threshold for interferon secretion upon viral infection [45].

As observed with other chronic viral infections, gut microbial dysbiosis is present in PLWH despite effective HIV viral control. This state is characterized by a lower diversity of gut microbiota composition, with a decrease of commensal bacterial taxa as *Lactobacilli* or Bacteroidaceae and enrichment of pathogenic taxa as Enterobacteriaceae [46,47,48]. Moreover, the beneficial mucin-degrading bacterial species *A. muciniphila*, of which abundance in the gut has been inversely associated with metabolic disorders and inflammation, was shown to be decreased in PLWH [49,50]. Altogether, increased relative abundance of inflammation-inducing bacteria in their host is believed to constitute a driver of systemic immune activation in PLWH. However, the lack of adjustment for confounding factors of most studies prevents the identification of a direct causal link [30].

Interestingly, CMV-induced microbiota changes have also been described, which is consistent with the fact that CMV widely infects the gut mucosa. A study from Gianella et al. examined colon biopsies from both PLWH and HIV-uninfected CMV-seropositive individuals. They reported that CMV detection in intestinal mucosa of PLWH, but not in HIV-uninfected controls, was associated with lower relative abundance of *Actinobacteria* [33]. Complex interactions between CMV and the microbiota have also been suggested by the study of Santos Rocha et al., evaluating the impact of subclinical viral infections on rhesus macaques [51]. In this model, experimental infection of specific-pathogen-free (SPF) macaques with rhesus CMV (rhCMV) resulted in microbiota changes, with a remarkable increase in abundance of butyrate-producing bacteria. Interestingly, in addition to its anti-inflammatory role, butyrate has also been shown to enhance the expression of CMV latent viral genes [52]. This intricate trans-kingdom interaction suggests a role for butyrate in the fine tuning of CMV subclinical replication maintenance.

Altogether, the presence of subclinical CMV replication in the gut and its influence on gut inflammation, microbial translocation and microbiota composition alteration is increasingly reported, both in PLWH and HIV-uninfected people. In healthy CMV-seropositive individuals, Gianella et al. detected CMV DNA in colon biopsies of 60.5% of the participants, irrespective of HIV infection [33]. Such detection was associated with higher levels of inflammatory cytokines (IL-6, IL-8, interferon-β [IFN-β]) in tissues of both PLWH and HIV-uninfected individuals, but resulted in a shift of microbiota composition only in PLWH [33]. Along the same line, we previously reported that CMV seropositivity was associated with gut inflammation in both PLWH and HIV-uninfected participants, but associated with microbial translocation and systemic inflammation in PLWH only (Figure 1) [17]. The negative impact of latent CMV infection thus seems to be potentialized in PLWH, representing double jeopardy to the health of these individuals.

## 5. Impact of CMV Infection on Response to Pathogens and Vaccines

Although difficult to assess due the heterogeneity of underlying comorbidities, age-induced impairment of both quantitative and qualitative immune system responses leads to a decreased response to vaccination [53]. With advancing age, total antibody titers decrease significantly [54], and the quality of these antibodies is also reduced [55]. Regarding the T-cell compartment, the number and immune repertoire of naïve T-cells available to respond to vaccine stimulation decreases with age as a result of thymic involution [56,57]. Moreover, accumulation of terminally differentiated cells, with a senescent phenotype and altered effector function is also a hallmark of aging.

Cytomegalovirus is partly responsible for the progressive inflation of the T-cell memory compartment, due to repeated antigen stimulation, however, its direct effect on response to pathogens and vaccines remains debated. Nevertheless, there is mounting evidence that CMV-seropositivity is associated with a reduced response to both invasion with a novel pathogen and to vaccination.

Influence of CMV in response to pathogens has been studied in mice, where CMV-specific CD8 T-cell expansion has been associated with a decreased T-cell repertoire available for response against other pathogens and decreased CD8 T-cell response upon influenza or West Nile virus superinfection [58]. However, this association is not consistently reported [59,60]. Again in a mouse model, immune activation due to recent infection with mCMV and other pathogens (murine γ-herpesvirus 68, influenza and helminth) was also associated with an decreased antibody response to yellow fever vaccine YF-17D [61]. In human studies, CMV-induced memory inflation was associated with a decreased memory response to EBV [62] and to influenza [63] in older people. Although not extensively studied yet, its role in the clinical course and severity of coronavirus disease 2019 (COVID-19) has also been proposed, due to the probable role of immunosenescence in the increased vulnerability of older patients [64,65].

The influence of CMV on the immune response to vaccination remains a matter of debate. In particular, many studies aimed to assess the influence of CMV in the immune response to influenza vaccination in the elderly, as the protection in this population remains unsatisfactory. In 2003, Trzonkowski et al. reported in 154 young and older individuals a negative correlation between responses to influenza trivalent inactivated vaccination (TIV) and anti-CMV IgGs, higher percentages of CD57^+^CD28^−^ lymphocytes, and higher circulating levels of TNF-α and IL-6 [66]. Derhovanessian et al. reported a negative association between CMV seropositivity and antibody titers after influenza immunization in an elderly population, but not in participants below 60 years of age [63]. In contrast, a study conducted by Wald et al. reported increased antibody titers only in CMV-seronegative vs. -seropositive participants below 60 years of age, whereas no difference could be observed in an older group [67]. A recent meta-analysis on the response rate to influenza vaccination revealed a trend for a decreased response in CMV-seropositive compared to CMV-seronegative participants [68]. Altogether, an impact of CMV on response to influenza vaccine can be assumed, although not unequivocally due to inconsistent reports. Larger and more systematic studies are needed to shed light on the influence of CMV latent infection on the influenza vaccine response.

Regarding other vaccines, a study evaluated the response to Ebola vaccine candidates (ChAd3-EBO-Z and MVA-EBO-Z) in healthy young adults in both the UK and Senegal [69]. CMV seropositivity was negatively associated with vaccine response in both UK and Senegalese cohorts, and correlated with an expansion of phenotypically senescent CD4 and CD8 T cells expressing CD57. Concerning vaccines against SARS-CoV-2, few PLWH have been included in the phase III vaccine trials, and constituted only 0.5% and 0.6% of participants in the Pfizer and Moderna trials, respectively [70]. Dedicated studies are thus urgently needed to evaluate the immunity after immunization against SARS-CoV-2 in PLWH.

Few studies have also been conducted in patients with comorbidities. In a recent study evaluating the response to vaccines in patients with chronic kidney disease, CMV seropositivity emerged as the stronger predictor of poor responsiveness to 23-valent pneumococcal polysaccharide PPV23 vaccination, rather than chronic kidney disease itself [71]. Conversely, in the same study, CMV seropositivity did not impact the response to trivalent inactivated influenza vaccine. A study also evaluated the response to influenza vaccination in patients with type 2 diabetes mellitus (T2DM), compared to healthy age-matched controls [72]. Whereas T2DM was not associated with a difference in response, CMV-seropositive participants responded surprisingly significantly better to vaccine than CMV-seronegative participants, in both healthy and diabetic participants.

Finally, in a proof-of-concept clinical trial, 36 CMV-seropositive patients with antineutrophil cytoplasmic antibody (ANCA)-associated vasculitis received either 6 months of valacyclovir or placebo [16]. Cytomegalovirus subclinical reactivation in these participants was associated with an impaired response to PCV13 pneumococcal vaccination. In comparison, antiviral suppression with valacyclovir could prevent reactivation events, decreased the abundance of CD4^+^CD2^−^ T-cells and increased vaccine response.

In infants with antenatal or postnatal CMV infection, studies have also evaluated the effect of CMV on response to vaccines, especially in poor-resource settings where the majority of children are infected before their first year of life [73]. CMV-induced alterations of immune response to measles and to a lesser extent to polio vaccines have been reported, whereas no difference in the response to Hib or tetanus vaccines were reported [74,75]. Interestingly, a study evaluating the immune response to oral polio vaccine in Zambian infants suggested a synergistic negative effect of HIV and CMV coinfection on the antibody response [76].

## 6. CMV as a Catalyzer of Immune Activation and Altered Response to Vaccine in PLWH

HIV-induced immune activation and premature immunosenescence compromise an adequate response to vaccination in PLWH [77,78]. By inducing direct accumulation of CMV-specific senescent t-cells and, and indirectly enhancing immune activation upon microbial translocation, CMV largely participates in the development of HIV-induced immunosenescence. As noted previously for systemic inflammation following gut inflammation and microbial translocation, CMV infection in PLWH might potentialize the intensity of immune activation and the subsequent alteration in vaccine response (Figure 1). Despite many indirect hints, evidence is however still lacking to prove this hypothesis. Understanding the underlying determinants of vaccine response in subpopulations is crucial in the fight against emerging pathogens, as illustrated by the ongoing global vaccination campaign against COVID-19 [70]. Dedicated comprehensive studies linking CMV/HIV co-infection and poor response to vaccines, which are still missing despite decades of research, are thus urgently needed.

## 7. Inhibiting CMV to Increase Response to Vaccines

Recent advances have been made in the fight against CMV and could help to distinguish its exact influence on health. Classical anti-CMV agents are associated with significant toxicities, as ganciclovir-mediated myelosuppression or foscarnet-associated nephrotoxicity, limiting their use to life-threatening clinically-significant CMV infections. However, the safe and effective anti-CMV drug letermovir, acting as a terminase inhibitor, has been recently developed and was approved by the FDA in 2017 for primary prophylaxis in adult CMV-seropositive allogeneic transplantation recipients [79,80,81]. Although the indications of this new drug remain strictly limited for now, the development of this new molecule may well be a game changer in the understanding and management of CMV infection. Other drug candidates include maribavir, a UL97 inhibitor currently in Phase III clinical trial for CMV infection treatment, and filociclovir, a UL54 inhibitor, which recently completed Phase I assessment [82,83].

Regarding anti-CMV vaccine development, despite decades of efforts and a place in the top priority list for vaccine development of the US Institute of Medicine, an effective vaccine is still missing. Many unsuccessful attempts have been made, with various methods (attenuated strains, viral vectored vaccines, subunit gB/pp65, lipid nanoparticles-encapsulated nucleoside-modified mRNA, peptides, enveloped virus-like particles) [84]. Although none of these studies led to a safe and effective vaccine, antigens required for immunizations have emerged and should guide further studies. In addition, the success of mRNA vaccines in the fight against COVID-19 helped to put in the pipeline few viruses lacking commercially available vaccines. A CMV mRNA vaccine from Moderna (mRNA-1647) is thus currently in phase II clinical trials, with promising interim data reported (NCT04232280).

The strong cellular and humoral immune responses against CMV, in addition to its large genome and its ability to superinfect a previously infected host, made this virus an attractive vaccine vector for other pathogens or neoplasia [85]. In rhesus macaques, several studies evaluated a fibroblast-adapted laboratory rhesus cytomegalovirus strain (RhCMV68-1) vector expressing simian immunodeficiency virus (SIV) proteins, and found an efficient but unconventional major histocompatibility complex E (MHC-E)-restricted CD8 T-cell response [86,87]. Preclinical studies altogether suggest that CMV-based vaccine vectors represent a promising approach in the quest for an efficient HIV vaccine, and a phase Ia, human study (NCT04725877), testing the VIR-1111 CMV-vectored HIV vaccine should shed further light on their future application.

## 8. Concluding Remarks

After decades of research, the complex interactions between coinfecting viruses, microbiota composition and immune activation should be considered to optimize HIV vaccine response. Although many open questions remain, mounting evidence highlights the capacity of CMV to profoundly shape the human immune system and impact the response to antigen encountering. This influence is even more prominent in PLWH, who already suffer from HIV-induced chronic immune activation and premature immunosenescence. In modulating vaccine immunogenicity, CMV has to be taken into consideration for vaccine development, but also when comparing trial outcomes between various populations exhibiting diverse CMV positivity rates. This is also relevant regarding HIV vaccines, due to the well-described effect of CMV and its high prevalence in PLWH. Recent epidemics of emerging pathogens such as Ebola or SARS-CoV-2 illustrate the crucial importance of an adequate response to a new antigen, should it be a virus or a new vaccine. Modulation of underlying causes of immune activation with targeted therapies prior to vaccination would constitute promising options to foster HIV vaccine development in the near future.

## Data Availability

Not applicable.
